# Comparisons of metastatic patterns of colorectal cancer among patients by age group: a population-based study

**DOI:** 10.18632/aging.101700

**Published:** 2018-12-28

**Authors:** Lin Yang, Xingli Yang, Wenzhuo He, Shousheng Liu, Chang Jiang, Kunqian Xie, Kunwei Peng, Yafei You, Bei Zhang, Liangping Xia

**Affiliations:** 1 Department of Medical Oncology, Sun Yat-sen University Cancer Center, State Key Laboratory of Oncology in South China, Collaborative Innovation Center of Cancer Medicine, Guangzhou, China; 2 Department of Radiotherapy, Sun Yat-sen University Cancer Center, State Key Laboratory of Oncology in South China, Collaborative Innovation Center of Cancer Medicine, Guangzhou, China; * Equal contribution

**Keywords:** metastatic colorectal cancer, age, sites of metastases, incidence of metastasis, prognosis

## Abstract

Population-based evaluations of the incidence of metastatic colorectal cancer at diagnosis among different age groups are lacking. Therefore, we investigated the effects of age at diagnosis on metastatic colorectal cancer and patients’ prognoses. The Surveillance, Epidemiology, and End Results database was used to identify patients diagnosed with metastatic colorectal cancer. Multivariate Cox regression analyses were performed to identify factors associated with poor survival. The Kaplan–Meier analysis was used to estimate survival differences between the subgroups. We identified 30,333 adult patients diagnosed with metastatic colorectal cancer between 2010 and 2014. The younger and middle-aged groups had better survival than the older group when brain metastasis was not involved. The liver was the most common site of metastasis followed by the liver and lung combined. Age at diagnosis was an independent factor in patients’ survival. Survival differences between two and three-sites of metastases were found in the middle-aged and older groups but not in the younger group. No survival differences between three and four sites of metastases were found in any of the age groups. Therefore, the incidence and prognosis of metastatic sites for metastatic colorectal cancer varied by age group.

## Introduction

Despite the improved survival of colorectal cancer (CRC) patients, metastatic disease still accounts for a high number of cancer-related deaths. Approximately 20% of patients present with metastatic disease at the time diagnosis [1]. The most common sites of CRC metastasis are the liver, lungs, and peritoneum, but there are other sites of metastasis, such as the bones, brain, and distant lymph nodes [2-5]. Autopsy studies have examined metastatic patterns and found that different primary cancers metastasize with different frequencies to different sites [5] and studies of CRC have revealed that histological subtypes influence metastatic patterns [5].

CRC predominantly occurs in the elderly population, and its incidence and mortality are expected to increase in this group [6]. Approximately 110,000 new cases of CRC were diagnosed in 2008 in Japan, and patients aged >65 years accounted for >70% of them [7]. However, among the patient population with distant metastasis of CRC, survival differences may depend on the site of the metastasis and the number of sites [8-10]. Little is known about the metastasis of this disease to different sites in different age groups [8,9,11,12].

Previous studies have provided evidence that the current combination chemotherapy regimens for metastatic colorectal cancer (mCRC) is tolerable for older persons with similar treatment benefits compared to younger patients [11]. However, the probability of older patients with mCRC being enrolled in clinical trials or receiving surgery is low [13] because older patients tend to be in poor physical condition [13].

This study used data from the Surveillance, Epidemiology, and End Results (SEER) cancer-registry program to identify individuals diagnosed with mCRC from 2010 to 2014 with the intent to gain insight into the relevance of age in the metastatic patterns of CRC in this population. Knowledge of metastatic distributions and survival differences among the age groups may help researchers devise clinical trials, especially, to make determinations regarding curative-intent interventions.

## RESULTS

### Demographic and clinical characteristics of patients with metastatic colorectal cancer by age group

Overall, 30,333 patients diagnosed with mCRC were included in our study, among which 4,309 (14.2%) were younger than 50 years old, 14,383 (47.2%) were between 50 and 69 years old, and 11,641 (38.4%) were older than 69 years old, they were defined as the younger (<50), middle-aged (50-69), and older groups (>69) respectively. There were 19,717 patients died at the end of the study and included 14,911 patients died from colorectal cancer. The percentage of deaths was 51.4% (2,215/4,309), 59.9% (8,615/14,383), and 76.3% (8,887/11,641) in the younger, middle-aged, and older groups, respectively. More detailed information about the age categories are presented in Table 1.

**Table 1:** Characteristics of patients with colorectal cancer with distant metastasis by age group.

**Patient characteristic**	**Total**	**<50 years**		**50-69 years**		**>69 years**		**P value**
	**No**	**No**	**%**	**No**	**%**	**No**	**%**	
**Sex**								<0.001
Male	16,616	2,350	54.5	8,455	58.8	5,811	49.9	
Female	13,717	1,959	45.5	5,928	41.2	5,830	50.1	
**Married**								<0.001
Unmarried	13,923	1,859	43.1	6,243	43.4	5,821	50	
Married	14,864	2,235	51.9	7,381	51.3	5,248	45.1	
Unknown	1,546	215	5	759	5.3	572	4.9	
**Race**								<0.001
Black	19,9130	2,402	55.7	9,052	62.9	8,456	72.6	
White	4,435	684	15.9	2,430	16.9	1,321	8.3	
Hispanic		750	17.4	1,648	11.5	982	8.4	
Asian/Pacific Islander	3,380	413	9.6	1,100	7.6	806	6.9	
Native American	2,319	47	1.1	119	0.8	66	0.6	
Unknown	232	13	0.3	34	0.2	10	0.1	
**Surgery**								<0.001
No	14,127	1,750	40.6	6,458	44.9	5,919	50.8	
Yes	16,139	2,553	59.2	7,897	54.9	5,689	48.9	
Unknown	67	6	0.1	28	0.2	33	0.3	
**T stage**								<0.001
Tis,T0,T1,T2 (0,1, 2,3)	3,961	568	13.2	1,854	12.9	1,539	13.2	
T3-T4 (4,5)	18,759	2,878	66.8	9,207	64	6,674	57.3	
Unknown	7,613	863	20	3,322	23.1	3,428	29.4	
**N stage**								<0.001
N0	9,607	1,116	25.9	4,382	30.5	4,109	42.8	
N1	9,569	1,506	35	4,817	33.5	3,246	27.9	
N2	7,760	1,306	30.3	3,761	26.1	2,693	23.1	
Unknown	3,397	381	8.8	1,423	9.9	1,593	13.7	
**Diagnosed methods**								<0.001
Biopsy	28,822	4,241	98.4	14,002	97.4	10,579	90.9	
Other method	1,511	68	1.6	381	2.6	1062	9.1	
**Pathology type**								<0.001
Adenocarcinoma	25,850	3,749	87	12,655	88	9,446	81.1	
Mucinous	2,558	429	10	1,157	8	972	8.3	
Other type	1,248	115	2.7	441	3.1	692	5.9	
Unspecified	677	16	0.4	130	0.9	531	4.6	
**Pathology grade**								<0.001
Well differentiated	1,204	165	3.8	586	4.1	453	3.9	
Moderately differentiated	15,152	2.308	53.6	7,527	52.3	5,317	45.7	
Poorly differentiated	5,924	918	21.3	2,767	19.2	2,239	19.2	
Undifferentiated	1,167	172	4	526	3.7	469	4	
Unknown	6,886	746	17.3	2,977	20.7	3,163	27.2	
**Positive lymph nodes**								<0.001
0	15,012	1,885	43.7	6,917	48.1	6,210	53.3	
1-3	415	63	1.5	183	1.3	169	1.5	
>4	14,472	2,293	53.2	7,060	49.1	5,119	44	
Unknown	434	68	1.6	223	1.6	143	1.2	
**Number of Lymph node**								<0.001
0	3,286	471	10.9	1,664	11.6	1,151	9.9	
<12	10,192	1,613	37.4	4,948	34.4	3,631	31.2	
>=12	1,763	327	7.6	829	5.8	607	5.2	
Unknown	15,092	1,898	44	6,942	48.3	6252	53.7	
**Tumor site**								<0.001
RCC	12,452	1,171	27.2	5,294	36.8	5,987	51.4	
LCC	8,797	1,550	36	4,331	30.1	2,916	25.0	
RSC	9,084	1,588	36.9	4,758	33.1	2,738	23.5	
**Tumor sizes**								<0.001
<=40 mm	6,649	1,002	23.3	3,159	22	2,488	21.4	
40-70 mm	9,867	1,442	33.5	4,803	33.4	3,622	31.1	
>=70 mm	4,341	656	15.2	2,124	14.8	1,561	13.4	
Unknown	9,476	1,209	28.1	4,297	29.9	3,970	34.1	

Abbreviations, RCC, right-sided colon cancer; LCC, left-sided colon cancer; RSC, rectosigmoid cancer.

Significant differences among the patient cohorts included race, tumor size, T stage, N stage, tumor location, degree of differentiation, and histological type (P< 0.001,respectively). Generally, the younger and middle-aged patients had larger tumors, advanced T stage, advanced N stage, more adenocarcinoma, and more moderate differentiation than the older patients did (P< 0.001).

Regarding to tumor location, the proportion of metastases were significantly different among age groups, for example, in RCC subgroup, the proportions were 27.2% in the younger cohort, 36.8% in the middle-age cohort, and 51.4% in the older-aged cohort. It indicated that the older patients with RCC had likely more metastases than the other groups. The younger and middle-aged patients had a significantly higher rate of surgery compared to their older counterparts (P< 0.001). The results may be attributed to their better physical condition to withstand the treatment. Specifically, African-American patients tended to have metastatic colorectal cancer at an older age (72.6%, 62.9%, and 55.7% in the older, middle-aged, and younger groups, respectively, P< 0.001). In contrast, White patients tended to be diagnosed with metastasis at a younger age (15.9%, 16.9%, and 8.3% in the younger, middle aged, and older groups, respectively).

### Different metastatic patterns of colorectal cancer in patients by age group

Many patients developed metastatic diseases in more than one site. Table 2 summarizes all possible combinations of four sites of metastases. We found the liver was the most common site of metastasis for colorectal cancer and accounted for more than half of all the sites in the three age groups (Table 2).

**Table 2:** The proportions of metastatic patterns in different age groups.

**Patient characteristics**	**Total**		**<50 years**		**50-69 years**		**>69 years**		**P value**
	**No**	**%**	**No**	***%***	**No**	***%***	**No**	***%***	
**Total**	30,333		4,309	14.2	14,383	47.4	11,641	38.4	
**One site**	18,833	62.1	2,690	62.4	8,901	61.9	7,242	62.2	<0.001
Bone only	327	1.1	53	1.2	141	1	133	1.1	
Lung only	1,913	6.3	219	5.1	820	5.7	1,874	16.1	
Liver only	16,478	54.3	2.405	55.8	7,880	54.8	5,193	44.1	
Brain only	115	0.4	13	0.3	60	0.4	42	0.4	
**Two sites**	5,367	17.7	694	16.1	2,709	18.8	1,964	16.9	0.158
Lung and liver	4,480	14.8	578	13.4	2254	15.7	1648	14.2	
Lung and bone	136	0.4	24	0.6	58	0.4	215	1.8	
Lung and brain	55	0.2	4	0.1	29	0.2	24	0.2	
Liver and bone	620	2	82	1.9	58	0.4	54	0.5	
Liver and brain	74	0.2	5	0.1	45	0.3	24	0.2	
Bone and brain	2	0.1	1	0.1	0	0	1	0.1	
**Three sites**	622	2.1	75	1.7	356	2.5	191	1.6	0.526
Lung, Liver and bone	501	1.7	59	1.4	279	1.9	163	1.4	
Lung, Liver and brain	89	0.3	11	0.3	57	0.4	21	0.2	
Liver, bone and brain	19	0.1	3	0.1	13	0.1	3	0.1	
Lung, bone and brain	13	0,1	2	0.1	7	0.1	4	0.1	
**Four sites**									
Liver, lung, bone and brain	31	0.1	2	0.1	21	0.1	8	0.1	
**Other sites**	5480	18.1	848	19.7	2396	16.7	2236	19.2	

Patients with multiple organ metastases had fewer treatment options and tended to have poorer outcomes. Unfortunately, at least 19.8% of all the cases had multiple organ metastases. The most common combination of organs with metastases was the liver and lung, which comprised 14.8% of patients with the multiple organ metastases. Only 31(0.1%) patients had metastases to all four sites (Table 2).

Significant differences in the rates of one site metastasis were found between the three age groups (P< 0.001). The older group tended to have more single lung metastasis (16.1%, 5.7%, and 5.1% in the older, middle-aged, and younger groups, respectively) and the younger group tended to have more single liver metastasis (44.1%, 54.8%, and 55.8% in the older, middle-aged, and younger groups, respectively). However, no differences were found in the rate of metastases to two (P = 0.158) or three sites (P = 0.526).

### Comparisons of OS

**Figure 1:**
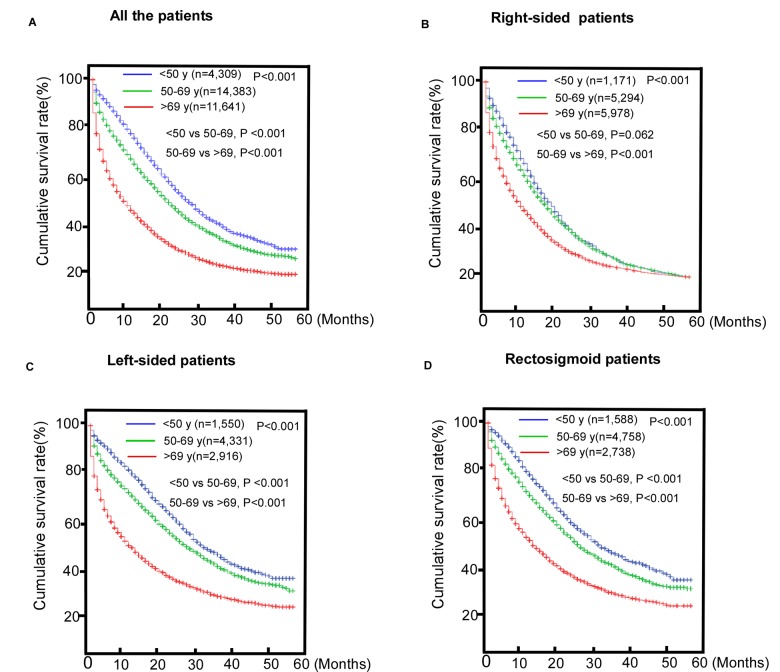
**Comparisons of survival of patients with metastasic colorectal cancer (mCRC).** (**A**) The entire cohort; (**B**) Right-sided colorectal cancer (RCC) subgroup; (**C**) Left-sided colorectal cancer (LCC) subgroup; (**D**) Rectosigmoid cancer (RSC) subgroup.

Substantive differences were found in overall survival (OS) (P< 0.001) between the three age groups (Figure 1). The older group had the worst survival with a median survival time (MST) of 6 months. The results of the survival analysis of the subgroups by tumor location (Figure 1) showed that the prognoses of patients with left-sided colon cancer (LCC) and recto-sigmoid cancer (RSC) worsened with increased age. The middle-aged and the younger groups with RCC had longer MSTs than the older group. However, the younger group’s prognosis was not as good as the middle-aged patients in the RCC group were (P >0.05).

**Figure 2:**
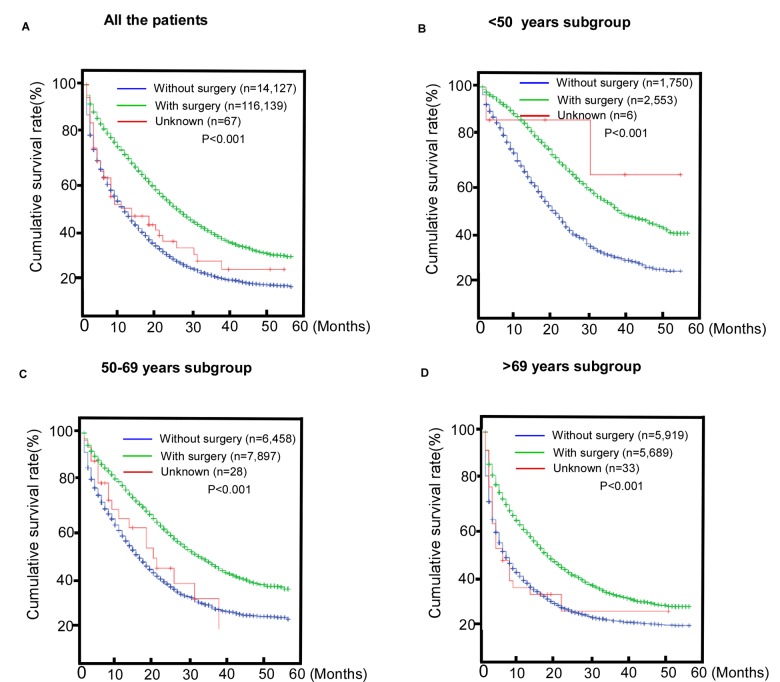
**Comparisons of the benefits of surgery for patients with metastatic colorectal cancer (mCRC)**. (**A**) The entire cohort; (**B**) <50 years old subroup; (**C**) 50-69 years old subgroup; (**D**) >69 years old subgroup.

We also found that patients who underwent surgery of the primary site or radiotherapy had better survival, indicating potential benefits from regional treatment for metastatic patients (Figure 2A, P< 0.001). The benefits observed in the subgroups are shown in Figure 2 B-D P< 0.001.

The results of the univariate analysis and multivariate Cox regression, which were conducted to evaluate the independent factors for OS are presented in Table 3. The univariate analysis showed that all the factors included in the study were associated with OS. The significant factors were then included in the multivariate analysis and all of the factors, except for marital status (P = 0.061), were associated with OS (P< 0.05). As shown in Table 3, age at diagnosis was an independent prognostic factor for patients with mCRC. Compared to the younger patients, the middle-aged patients had worse OS (HR: 1.389, 95% CI: 1.230–1.351, P < 0.001) and the older patients had the worst OS (HR: 2.141, 95% CI: 2.041–2.247, P < 0.001).

**Table 3:** Univariate and multivariate analyses of overall survival.

Patient characteristics	**Univariate analysis**		**Multivariate analysis**	
**OS**		**OS**	
	**HRs (95%CI)**	**P-value**	**HRs (95%CI)**	**P-value**
**Sex**				
Female vs Male	1.042 (1.014-1.072)	0.004	0.943 (0.916-0.971)	<0.001
**Age group**		<0.001		<0.001
<50	Reference		Reference	
50-69	1.334 (1.273-1.398)	<0.001	1.389 (1.230-1.351)	<0.001
>69	2.428 (2.317-2.545)	<0.001	2.141 (2.041-2.247)	<0.001
**Married**		<0.001		0.061
Unmarried	Reference		Reference	
Married	1.180 (1.115-1.311)	<0.001	1.138 (1.109-1.168)	<0.001
Unknown	0.867 (0.845-0.890)	<0.001	0.908 (0.885-0.932)	<0.001
**Race**				<0.001
Black	Reference		Reference	
White	1.072 (1.030-1.115)	0.001	1.099 (1.055-1.144)	<0.001
Hispanic	0.895 (0.855-0.938)	<0.001	1.001 (0.955-1.050)	0.953
Asian/Pacific Islander	0.862 (0.816-0.911)	<0.001	0.944 (0.893-0.998)	0.041
Native American	1.042 (0.899-1.222)	0.612	1.262 (1.075-1.480)	0.004
Unknown	0.575 (0.371-0.892)	0.013	0.740 (0.477-1.148)	0.179
**Surgery**		<0.001		<0.001
No	Reference		Reference	
Yes	0.472 (0.458-0.485)	<0.001	0.694 (0.641-0.752)	<0.001
Unknown	0.835 (0.629-1.109)	0.214	0.837 (0.629-1.113)	0.220
**T stage**		<0.001		<0.001
Tis,T0,T1,T2 (0,1, 2,3)	Reference		Reference	
T3-T4 (4,5)	0.737 (0.706-0.769)	<0.001	1.051 (1-1.105)	<0.001
Unknown	1.442 (1.377-1.510)	<0.001	1.080 (1.030-1.134)	0.002
**N stage**		<0.001		<0.001
N0	Reference		Reference	
N1	0.775 (0.748-0.804)	<0.001	0.900 (0.862-0.940)	<0.001
N2	0.826 (0.796-0.857)	<0.001	1.083 (1.021-1.149)	0.008
Unknown	1.570 (1.501-1.642)	<0.001	1.1080 (1.030-1.133)	0.002
**Diagnosed methods**				
Other method vs Biopsy	3.345 (3.163-3.537)	<0.001	1.401 (1.297-1.513)	<0.001
**Pathology type**		<0.001		<0.001
Adenocarcinoma	Reference		Reference	
Mucinous	1.163 (1.107-1.222)	<0.001	1.154 (1.097-1.215)	<0.001
Other type	2.507 (2.356-2.669)	<0.001	1.489 (1.390-1.595)	<0.001
Unspecified	4.422 (4.079-4.793)	<0.001	1.761 (1.588-1.953)	<0.001
**Pathology grade**		<0.001	1.401 (1.297-1.513)	<0.001
Well differentiated	Reference		1.000	
Moderately differentiated	0.969 (0.897-1.047)	0.426	1.081 (1-1.168)	0.050
Poorly differentiated	1.542 (1.424-1.671)	<0.001	1.602 (1.477-1.737)	<0.001
Undifferentiated	1.594 (1.441-1.764)	<0.001	1.748 (1.577-1.937)	<0.001
Unknown	1.989 (1.837-2.153)	<0.001	1.246 (1.148-1.351)	<0.001
**Positive lymph nodes**		<0.001		<0.001
0	Reference		Reference	
1-3	0.667 (0.592-0.751	<0.001	1.324 (1.137-1.542)	<0.001
>4	0.480 (0.467-0.495)	0.791	0.792 (0.713-0.881)	<0.001
Unknown	0.648 (0.577-0.728)	<0.001	0.902 (0.794-1.024)	0.110
**Number of Lymph node**		<0.001		<0.001
0	Reference		Reference	
<12	1.408 (1.330-1.491)	<0.001	1.356 (1.264-1.456)	<0.001
>=12	2.194 (2.035-2.365)	<0.001	1.764 (1.608-1.935)	<0.001
Unknown	2.849 (2.698-3.009)	<0.001	1.541 (1.379-1.721)	<0.001
**Tumor site**		<0.001		<0.001
RCC	Reference		Reference	
LCC	0.715 (0.691-0.739)	<0.001	0.805 (0.777-0.834)	<0.001
RSC	0.706 (0.683-0.730)	<0.001	0.642 (0619-0.666)	<0.001
**Tumor sizes**		<0.001		<0.001
<=40 mm	Reference		Reference	
40-70 mm	1.147 (1.101-1.194)	<0.001	1.116 (1.071-1.162)	<0.001
>=70mm	1.384 (1.318-1.453)	<0.001	1.274 (1.213-1.338)	<0.001
Unknown	1.857 (1.785-1.932)	<0.001	1.163 (1.112-1.216)	<0.001
**Metastasis organ number**		<0.001		
1	Reference		Reference	
2	2.413 (1.642-3.546)	<0.001	2.281 (1.551-3.353)	<0.001
3	1.481 (1.428-1.535)	<0.001	1.274 (1.228-1.322)	<0.001
4	2.049 (1.873-2.241)	<0.001	1.680 (1.535-1.839)	<0.001
Other organ metastasis	0.958 (0.923-0.996)	0.029	0.905 (0.869-0.941)	<0.001

Abbreviations, OS, overall survival; RCC, right-sided colon cancer; LCC, left-sided colon cancer; RSC, Rectosigmoid cancer.

HRs: hazard ratios; CI: confidence interval.

### Comparisons of survival between patients with single and multi-site metastatic colorectal cancer

**Figure 3:**
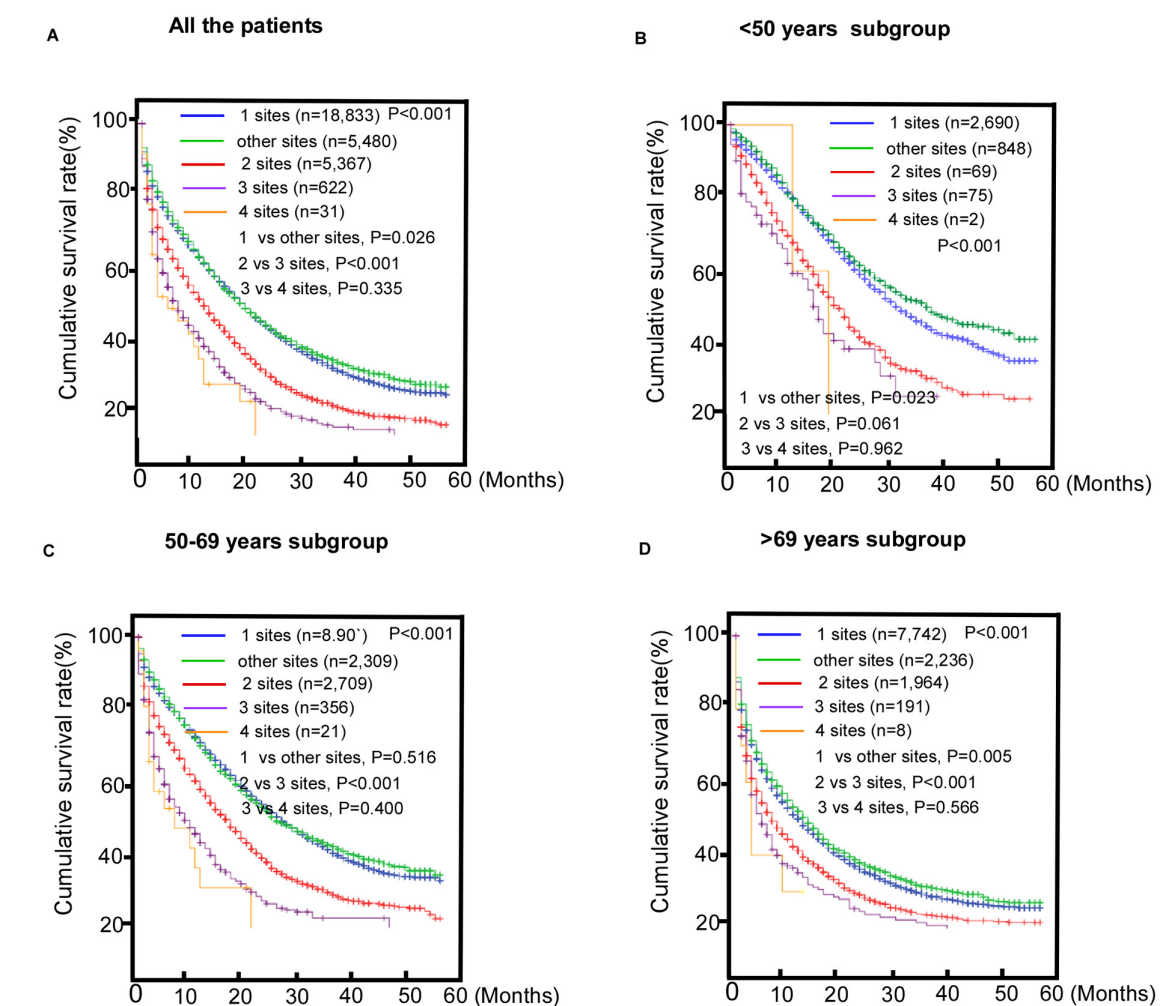
**Comparisons of survival among patients with metastatic colorectal cancer (mCRC) with single or multi-site metastases.** (**A**) The entire cohort; (**B**) < 50 years old subgroup; (**C**) 50-69 years old subgroup; (**D**) >69 years old subgroup.

We compared the effects of CRC metastases to single and multiple distant organs on the MST of the study population. The results indicated that there were significant differences in OS among the patients with different specific metastatic sites (Figure 3). No survival difference was found between patients with metastases to three or four sites (P = 0.335, Figure 3A). This survival difference between subgroups is shown in Figure 3B-D. However, no survival difference was found between patients with metastases to two or three sites in the younger group (P = 0.061). Among the patients in the middle-aged group, no survival difference was found between those with single site metastasis and metastasis to other sites (P = 0.516).

Among the patients with single site metastasis, those with lung metastasis only, had a significantly longer survival compared to the other metastatic patients (MST = 18 months, Table 4). Patients with liver invasion only, had a similar intermediate MST of 15 months. However, the brain metastasis only group (MST = 6 months) and bone metastasis only group (MST = 6 months) had the poorest prognosis compared to the other groups. Among the patients with multi-site metastases, those with lung and liver metastasis had the best survival (MST = 10 months) and patients with lung and bone metastasis had intermediate length of survival (MST = 8 months). However, the survival of the patients with other combinations of sites was very poor and no significant differences were found.

**Table 4:** The survival analyses of metastatic patterns in different age groups.

**Patient characteristics**	**Total**	**<50 years**	**50-69 years**	**>69 years**	**P value**
	**MST (mo)**	**MST (mo)**	**MST (mo)**	**MST (mo)**	
**Total**	13 (12.69-12.3)	23 (22.08-23.92)	17 (16.51-17.49)	6 (5.7-6.3)	<0.001
**One sites**	15 (14.57-15.43	25 (22.38-26.25)	20 (19.33-20.67	7 (6.6-7.4)	<0.001
Bone only	6 (4.55-7.44)	12 (8.49-15.51)	10 (6.08-13.92)	2 (1.12-2.88)	<0.001
Lung only	18 (16.58-19.41)	30 (25.77-34.23)	25 (21.40-28.6)	10 (8.26-11.64)	<0.001
Liver only	15 (14.55-15.45)	25 (23.74-26.26)	20 (19.31-20.69)	7 (6.59-7.41)	<0.001
Brain only	6 (4.18-7.8)	13 (1.88-24.12)	8 (5.23-10.77)	4 (2.06-5.94)	<0.001
**Two sites**	9 (8.46-9.54)	16 (14.55-17.45)	12 (11.21-12.79)	4 (3.58-4.42)	<0.001
Lung and liver	10 (9.37-10.63)	17 (15.28-18.73)	13 (12.1-13.9)	4 (3.52-4.84)	<0.001
Lung and bone	8 (5.27-10.73)	13 (2.34-23.66)	10 (6.08-13.92)	4 (2.28-5.73)	0.001
Liver and bone	5 (3.9-6.08)	10 (7.62-12.38)	10 (6.08-13.92)	3 (2.46-3.54)	<0.001
Lung and brain	4 (1.2-6.78)	8 (3.42-11.21)	7 (3.96-10.04)	2 (1.31-2.69)	0.062
Liver and brain	3 (1.33-4.67)	5 (1.08-8.92)	3 (1.84-4.17)	1 (0.04-1.96)	0.079
Bone and brain	4	4		7	0.317
**Three sites**	3 (1.7-4.3)	12 (7.7-16.3)	5 (3.91-6.09)	2 (0.15-3.85)	<0.001
Lung, Liver and bone	5 (3.92-6.08)	12 (6.88-17.12)	5 (3.8-6.1)	3 (2.17-3.83)	<0.001
Lung, Liver and brain	6 (3.54-8.46)	8 (5.51-10.45)	6 (4.5-7.49)	2 (0.27-3.73)	0.081
Liver, bone and brain	3 (1.1-8.17)	3 (1.2-6.2)	6 (3.2-8.79)	1	0.060
Lung, bone and brain	3 (1.33-4.67)	6	3 (1.83-4.17)	1	0.999
**Four sites**					
Liver, lung, bone and brain	3 (1-8.17)	12 (8.64-10.34)	3 (1.2-7)	2 (0.15-3.85)	0.420
**Other sites**	15 (1.24-15.76)	27 (24.01-29.99)	19 (17.67-20.33)	8 (7.14-8.86)	<0.001

Abbreviations, MST, median survival time.

The prognoses of patients in the three age groups with the same metastatic patterns were analyzed and the results showed that their prognoses worsened with increased age among the patients with single site metastasis. Among the patients with multiple metastases, those in the younger and middle-aged groups had better survival than the patients in the older group when brain metastasis was not involved. In other words, when patients with multiple metastases had brain metastasis, there were no survival differences among the three age groups.

## DISCUSSION

This large-scale study provided more in-depth knowledge and a better understanding of the heterogeneity of colorectal cancer among different age groups. We found that a younger age at diagnosis was associated with being White, having advanced N stage, more lymph node involvement, and a tendency to have LCC. Patients in the younger age group were more likely to have single liver metastasis, but less likely to have single lung metastasis compared with patients in the older age group, and the younger patients had better survival when brain metastasis was not involved.

The most common site of CRC metastasis is the liver, which is consistent with the results of a previous study [14], and the mechanism is thought to be multifaceted. First, the “seed and soil hypothesis” may partially account for the phenomenon of different metastatic patterns [15]. It seems that tumor cells from different subpopulations have favored microenvironments in distant organs, which facilitate their optimal invasion and proliferation in these organs. Second, a study revealed that neutrophils contribute to the colonization of breast cancer cells in the lung [16]. The existence of a similar mechanism for circulating tumor cells in the metastasis of CRC to the liver should be studied further. Third, previous evidence indicates that venous drainage of the colon to the portal system might be a potential mechanism underlying the metastatic pattern of CRC to the liver, first, and then to the lungs [17,18]. The underlying molecular mechanisms need further investigation to yield findings that may be used for cancer prevention.

Consistent with previously published reports [19-21], this study revealed a significant difference in OS between patients in the older age group and their younger counterparts. Age at diagnosis was one of the independent prognostic factors in the study population. The MST was 23 months for the younger patients and 17 and 6 months for the middle-aged and older groups, respectively, which may have a multi-factorial explanation. Many age-related factors, such as lower immune response [22], and higher levels of chronic inflammation [23] may influence survival of metastasis. Hu et al., reported that patients younger than 50 years have a better chance than those older than 50 years of receiving chemotherapy, radiation therapy, and surgery [24,25]. However, elderly patients are less likely to receive optimal treatment because of age-related increases in the deterioration of organ function or comorbidities [26]. Attenuation of the immune system has also been reported to influence (i.e., worsen) survival in older adults with mCRC.

Interestingly, the survival time of the younger group of patients with RCC was similar to that of the middle-aged patients. Other studies have suggested differences in terms of genetics, biology, and demographics between tumor locations of CRC; RCC was more common for both the older groups [27,28]. A recent molecular subtype analysis of CRC patients showed that the “type 5” group (MSI-high, BRAF-and KRAS-mutation negative, non-CpG isl and methylator phenotype) was present in a significant portion of patients aged < 40 years and 40–49 years (10% and 20%, respectively) [29].

Additionally, younger patients presented with metastatic disease at the time of diagnosis more often than did older patients. This may be because RCC is more likely to be classified as a mucinous adenocarcinoma (MC) in older adults [27,28], and MC has been reported to be more frequent than adenocarcinoma (AC) in presentations of multi-metastatic diseases [5]. The poor prognosis of the RCC patients with metastatic disease might have been due the fact that curative surgery is often limited to patients with liver metastasis [11,30]. This might have led to the similar survival times of the younger and the middle-aged groups with RCC. Nevertheless, there might have been other underlying factors. More importantly, we found that patients with different metastatic patterns had different survival outcomes. Specifically, the liver metastasis only group had the longest MST compared to the other patients with metastases, whereas the brain metastasis only group and the multi-site metastases group had the poorest outcomes. Despite their large numbers, neither clinical trials nor germinate immunotherapy-based treatments have shown significant improvements in patients with metastases [31,32]. Unfortunately, therapies are limited for patients with brain metastasis (mainly because of the blood-brain barrier) and multi-site metastases [33]. These results call for greater efforts to improve precision in medicine for the prevention and treatment of CRC metastasis on an individual basis.

This study has some limitations. We could not collect detailed information about the patients’ treatment, such as surgical procedures, chemotherapy regimens, or radiation methods from the SEER database, which may be a confounding factor in the results. Several other factors, other than age at diagnosis, might have also influenced the survival time of patients with mCRC. Therefore, the results need further validation in future studies.

## CONCLUSION

Our research summarized the tumor characteristics and survival outcomes of patients in three age groups with mCRC from a large sample of the population. Age was a robust prognostic factor and patients in the younger age group were more likely to have single liver metastasis, but less likely to have single lung metastasis compared with the patients in the older age group. The younger patients had better survival when their cancer did not involve brain metastasis. To determine more appropriate healthcare for aging patients with mCRC, further investigations of biochemical and molecular changes with aging are required.

## MATERIALS AND METHODS

Data were retrieved from the SEER database between 2010 and 2014. The datasets, which are available in the SEER dataset repository at: https://seer.cancer.gov/, represented 30% of the United States population. Pathology was classified as adenocarcinoma (AC), mucinous adenocarcinoma (MC), or other. Grade was defined as well differentiated, moderately differentiated, and poorly differentiated, or undifferentiated. Tumor location and their anatomical components, including RCC were classified as follows: RCC (cecum, ascending colon, hepatic flexure, and transverse colon), LCC (splenic flexure, descending colon, and sigmoid colon), RSC (recto-sigmoid junction and rectum), and appendix cancer [34]. Race/ethnicity was categorized as previously described [35]. The SEER 18 dataset categorized ethnicity as White, African-American, Native American/Alaskan Native, Asian/Pacific Islander, and unknown. The presence of bone, lung, liver, and brain metastases at diagnosis were available in the SEER database and were categorized as the number of metastases among the patients in our study. Patients were observed after the first diagnosis of CRC until the last follow-up, death, or end of the study, whichever occurred first.

### Statistical analysis

The Chi square test was used for the comparisons of categorical variables and the Kaplan-Meier method was used to estimate survival differences between the subgroups. Univariate and multivariable Cox regression analyses were performed to identify covariates associated with poor survival. All statistical tests were two-tailed and P< 0.05 was considered statistically significant. Statistical analyses were performed using SAS 9.2 (SAS Institute, Cary, NC, USA).

### Assessing locus of control

Women completed a condensed version of the Adult Nowicki Strickland Internal External control scale (ANSIE) [24] in questionnaires administered at mean ages 30 and 48 years. The original ANSIE comprises 40 items in a yes/no format, which assess perceived control. The version used in the present study comprises 12 of the original 40 items, which were chosen after factor analysis of the ANSIE administered as a pilot to

### Availability of data and material

All data were retrieved from the Surveillance, Epidemiology, and End Results (SEER) program of the National Cancer Institute between 2010 and 2014. The datasets are available in the SEER dataset repository https://seer.cancer.gov/.
